# Spatial patterns of neuronal activity in rat cerebral cortex during non-rapid eye movement sleep

**DOI:** 10.1007/s00429-014-0867-9

**Published:** 2014-08-13

**Authors:** Tim Wanger, Wolfram Wetzel, Henning Scheich, Frank W. Ohl, Jürgen Goldschmidt

**Affiliations:** 1 Department Systems Physiology of Learning, Leibniz Institute for Neurobiology (LIN), Brenneckestraße 6, 39118 Magdeburg, Germany; 2 Emeritus Group Lifelong Learning, Leibniz Institute for Neurobiology (LIN), Brenneckestraße 6, 39118 Magdeburg, Germany; 3 Otto-von-Guericke University, 39106 Magdeburg, Germany; 4 Center for Behavioral Brain Science (CBBS), Magdeburg, Germany

**Keywords:** Cortical columns, Infragranular layers, Laminar microcircuits, Slow-wave sleep, Thallium-autometallography

## Abstract

It is commonly assumed that cortical activity in non-rapid eye movement sleep (NREMS) is spatially homogeneous on the mesoscopic scale. This is partly due to the limited observational scope of common metabolic or imaging methods in sleep. We used the recently developed technique of thallium-autometallography (TlAMG) to visualize mesoscopic patterns of activity in the sleeping cortex with single-cell resolution. We intravenously injected rats with the lipophilic chelate complex thallium diethyldithiocarbamate (TlDDC) during spontaneously occurring periods of NREMS and mapped the patterns of neuronal uptake of the potassium (K^+^) probe thallium (Tl^+^). Using this method, we show that cortical activity patterns are not spatially homogeneous during discrete 5-min episodes of NREMS in unrestrained rats—rather, they are complex and spatially diverse. Along with a relative predominance of infragranular layer activation, we find pronounced differences in metabolic activity of neighboring neuronal assemblies, an observation which lends support to the emerging paradigm that sleep is a distributed process with regulation on the local scale.

**Electronic supplementary material:**

The online version of this article (doi:10.1007/s00429-014-0867-9) contains supplementary material, which is available to authorized users.

## Introduction

Sleep is an almost ubiquitous phenomenon in the animal kingdom and is defined as a homeostatically regulated state of immobility, reduced arousal and rapid reversibility (Siegel 2005; Cirelli and Tononi 2008). In addition to its purported restorative functions (Walker et al. 1979; Dworak et al. 2010), sleep has been reported to be beneficial or facilitatory in the process of memory consolidation and retention (e.g. Jenkins and Dallenbach 1924; Gais et al. 2000; Fenn et al. 2003; Wetzel et al. 2003; Huber et al. 2004; Hennevin et al. 2007; Ji and Wilson 2007; Diekelmann et al. 2011; Stickgold and Walker 2013). In particular, episodes of non-rapid eye movement sleep (NREMS) have been proposed to be critically involved in cortical plasticity processes, i.e. in the functional reorganization and/or maintenance of neuronal connections (Sejnowski and Destexhe 2000; Steriade and Timofeev 2003; Tononi and Cirelli 2006; Wang et al. 2011; Chauvette et al. 2012). Moreover, NREMS has been used as a tool to study the neural correlates of the transition from awake states to environmentally disconnected states in humans (Massimini et al. 2005; Horovitz et al. 2009; Larson-Prior et al. 2009), thus providing mechanistic insight into the biology of fading and diminished awareness.

While extensive research has been done on the physiological properties of NREMS (e.g. Steriade and McCarley 2005; Buzsaki 2006), the spatial organization of cortical activity during NREMS and, in particular, to which degree it differs from wakefulness (WK) remains largely unexplored. This shortcoming is, for the most part, due to methodological difficulties. Functional MRI in sleeping animals is complicated by scanner noise, while the spatial resolution of positron emission tomography (PET) is insufficient to image activity patterns on the level of cortical cell assemblies. The 2-deoxyglucose (2-DG) technique has been applied to map local cerebral glucose utilization during NREMS in primates (Kennedy et al. 1982) and rodents (Ramm and Frost 1983), but this method requires a postinjection period of 45 min for the tracer to be cleared from circulation which confounds mapping of discrete, short-duration sleep stages in small animals. Expression patterns of immediate early genes (IEGs), such as zif-268 (Ribeiro et al. 2002) and c-Fos (Gvilia et al. 2006; Lu et al. 2006; Gerashchenko et al. 2008), have been used to study neural activity during sleep, but the link between discrete sleep phases and post-sleep IEG expression is ambiguous. Furthermore, IEG expression is not suited to map activity under constant, non-stimulus conditions, where no changes in external or internal input are provided (Kovács 2008), which makes it difficult to investigate spontaneous activity patterns during continuous NREMS episodes with this technique.

Here we used thallium-autometallography (Tl-AMG), a method developed for single-cell resolution mapping of neuronal and astrocytic potassium uptake (Goldschmidt et al. 2004, 2010), to analyze the spatial patterns of neuronal activity during discrete (5 min) and spontaneous NREMS episodes of adult rats. Tl-AMG is a tracer technique similar in rationale to the 2-DG method, but is based on the fact that neuronal transmembrane movements of potassium (K^+^) increase with increasing activity (Keynes 1951; Keynes and Ritchie 1965). K^+^-analogus like the univalent thallium ion Tl^+^ can be used, therefore, as tracers for imaging neuronal activity (Landowne 1975; Goldschmidt et al. 2004). Furthermore, we have recently shown that the lipophilic chelate complex thallium diethyldithiocarbamate (TlDDC) passes the blood–brain barrier and can be used for mapping neuronal activity with single cell resolution in freely moving, behaving rodents, with short stimulation times of 5 min (Macharadze et al. 2009, 2012; Goldschmidt et al. 2010; Wanger et al. 2012; Lison et al. 2013; Stöber et al. 2013).

Our results show that regional Tl^+^-uptake rates in various cortical fields do not differ between WK and NREMS when compared to global Tl^+^-uptake within each state. Laminar Tl^+^-uptake in NREMS within both unimodal and multimodal cortical fields was significantly higher in infragranular layers, and distinct changes in laminar uptake profiles were found between NREMS and WK for primary auditory cortex (A1) and retrosplenial association cortex (RSC). Interestingly, large parts of the neocortex showed pronounced columnar Tl^+^-uptake patterns in NREMS, which is supportive of the view that NREMS is regulated on a local level of cortical assemblies (Krueger and Obál 1993; Krueger et al. 2008).

## Materials and methods

All experiments were in compliance with the guidelines of the European Community (EUVD 86/609/EEC) and were approved by an ethics commission of the state of Sachsen-Anhalt.

### Animals

Forty adult male Wistar rats (250–400 g) were used for the experiments described here. Animals were housed under standard laboratory conditions in a room with a 12 h dark/12 h light cycle, with lights on at 7:00. Ambient temperature in the room was regulated at 23 °C. Ambient temperature during the experiment was regulated between 22 and 24 °C.

### Surgery

Animals were induced by intraperitoneal infusion of pentobarbital (50 mg/kg, Nembutal, Sigma). Further anesthetic was given as necessary to maintain areflexia. After onset of anesthesia, the right external jugular vein was catheterized as described previously (Goldschmidt et al. 2010). Silicone rat jugular vein catheters (Alzet, Charles River) were implanted sterilely in 25 animals. For 15 other animals, jugular catheters were custom made from 10 cm of polyethylene tubing (PE 50, Braintree Scientific) with a 1-cm cuff at the tip consisting of silicon tubing (RenaSIL 065, Braintree Scientific). Catheters were filled with heparinized saline and flushed every 3 days.

After the catheterization procedure, animals were implanted with electrocorticogram (ECoG) and neck-muscle electromyogram (EMG) electrodes as described previously (Wetzel et al. 1994, 2003). In short, two stainless steel screws (winding diameter 1 mm, length 2 mm, FST) connected to Teflon-coated stainless steel wires (diameter 0.5 mm, Science Products) were implanted on the cortical surface of the right hemisphere (bregma: −2 mm, lateral: 2 mm and bregma: −6.5 mm, lateral: 2 mm) and served as ECoG-electrodes. Three uncoated and three Teflon-coated stainless steel wires (diameter 0.17 mm, Science Products) were implanted into the neck-musculature and served as EMG-electrodes.

Animals were allowed to recover from surgery for a minimum of 5 days prior to experiments.

### Polygraphic recordings

**Fig. 1:**
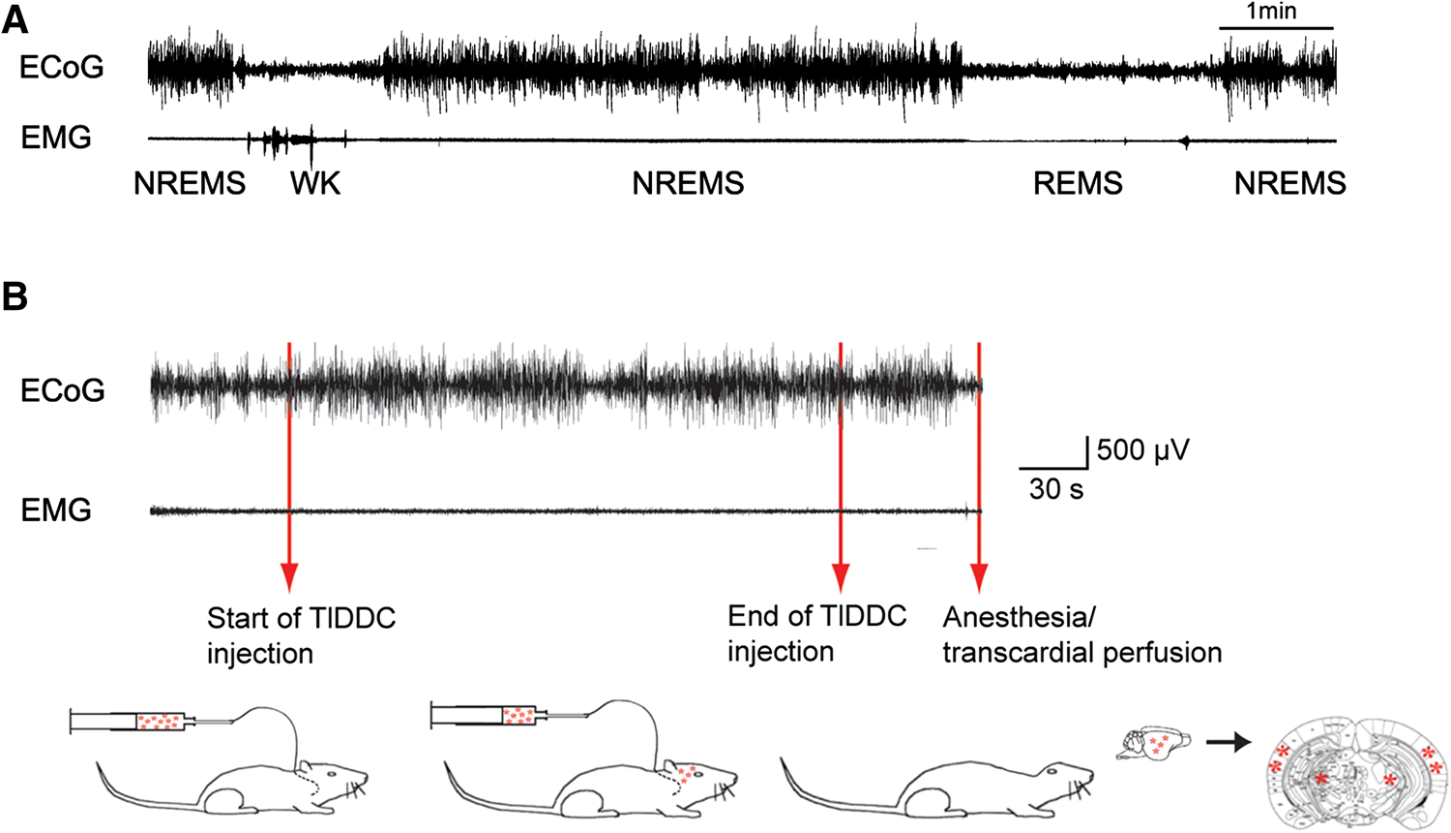
**a** Example of a typical sleep-wake recording. Episodes of non-rapid eye movement sleep (NREMS), wakefulness (WK) and rapid eye movement sleep (REMS) were classified according to combined electrocorticogram (ECoG) and electromyogram (EMG) traces. Modified after Wetzel et al. (2003). **b** Intravenous injection of thallium diethyldithiocarbamate (TlDDC) during NREMS. TlDDC was continuously infused during 4 min of stable NREMS, before TlDDC infusion was discontinued. One minute later, animals were given an overdose of anesthetic and were transcardially perfused. Autometallographic processing of the brain was performed as described in Methods to allow for light microscopic visualization of Tl^+^-uptake patterns. For the WK group, TlDDC injections were performed in a similar fashion during stable episodes of WK

Experiments were conducted in a sound chamber (Industrial Acoustics Company) with a window through which to visually monitor animal behavior. Animals were habituated to the experimental setup during the first week after surgery. ECoGs and EMGs were recorded via an 18-channel recording system (Model 4418G, Nihon Kohden) and evaluated visually using standard criteria (Fig. 1a). For ten animals, recordings were additionally digitized on a PC running custom data acquisition software written in Matlab (Mathworks). ECoG recordings were referenced through the EMG electrodes. For EMG recordings, electrodes were referenced against each other in a bipolar manner. Electrophysiological signals were filtered according to the signal (ECoG: 0.3–35 Hz; EMG: 160–1,000 Hz).

### Intravenous application of Tl^+^ during NREMS and WK

The tracer ion thallium (Tl^+^) was injected in the form of the electroneutral lipophilic chelate complex thallium diethyldithiocarbamate (TlDDC). This complex crosses the blood–brain barrier and releases Tl^+^ prior to neuronal or glial uptake. TlDDC can thus be used for high-resolution mapping of neuronal activity and offers substantial advantages over the injection of thallium salts like thallium (I) acetate (TlAc) (Goldschmidt et al. 2010; Wanger et al. 2012).

On the day of the experiment, the implanted catheter was connected to an extension tube (PE 50, length 80 cm) filled with saline. The extension tube led outside of the sound chamber and connected to a 2-ml syringe mounted on a microinjector (CMA 100 Microinjection Pump, Carnegie Medicine) via a 23-G needle.

For NREMS experiments, polygraphic recordings always started at 8 am local time and vigilance states were classified as described previously (Wetzel et al. 2003). As periods of NREMS became longer and more prevailing, a 2-ml syringe was filled with freshly prepared 0.05 % TlDDC-solution in 0.9 % NaCl and was then mounted on the microinjector and attached to the PE50-extension. If a NREMS phase was stable for about 1 min, injection of TlDDC was started with a speed of 250 µl/min and a final volume of 1 ml TlDDC was continuously injected over 4 min (Fig. 1b). Injection times for TlDDC ranged from 8 am to 11 am for the NREMS group, i.e. 1–4 h after lights on. After 4 min, TlDDC injection was discontinued and the PE-50 extension tube was reattached to the 2 ml syringe filled with saline to clear the catheter and extension tube from remaining TlDDC. One minute after clearance of the PE-50 tubing from TlDDC, animals were overdosed by an intravenous bolus-injection of ketamine (25 mg per kg BW, Ketamin-ratiopharm), and were transcardially perfused as described below. For WK experiments, the start of polygraphic recordings was shifted to 6 pm, to account for the nocturnal rhythm of the animals. Classification of WK phases and TlDDC injection was performed as described above. Injection times for TlDDC ranged from 6 pm to 7 pm for the WK group.

Further analysis was performed only on animals that showed a consistent polygraphic state-as defined by more than 80 % NREMS or WK, respectively, from the start of the TlDDC injection until the induction of anesthesia. As NREMS phases in rats are usually in the order of 30 s–10 min, with approximately 50 % of all NREMS episodes being shorter than 2 min and 80 % being shorter than 4 min (Suppl. Figure 1), polygraphic state changes occurred in 80 % of the animals during the critical 5-min period of TlDDC exposure. Given that the tracer Tl^+^ redistributes as a function of time (Wanger et al. 2012), tracer injection cannot simply be discontinued and resumed at the onset of the next NREMS phase, as this would lead to a blurred metabolic image confounded by factors other than NREMS (Wanger et al. 2012). This, and the fact that the onset of the TlDDC injection triggered a state transition from NREMS to WK in a small subset of animals, resulted in a high exclusion rate within the NREMS group. Eventually, six animals fulfilled the criteria to be classified as NREMS animals, whereas five animals constituted the WK group.

### Transcardial perfusion, tissue processing and staining

Transcardial perfusion, tissue processing and staining were performed as described in detail previously (Goldschmidt et al. 2004, 2010). Animals were perfused with a sodium sulfide-solution (0.325 % Na_2_S in 100 mM phosphate buffer, pH 7.4) and a sulfide-glutaraldehyde solution (0.16 % Na_2_S and 3 % glutaraldehyde in 100 mM phosphate buffer, pH 7.4). Approximately 90 s after the onset of anesthesia, surgery for transcardial perfusion was completed, and precipitation of Tl^+^ by sodium sulfide-solution started.

After perfusion, brains were removed and cryoprotected for 48 h in 30 % sucrose in 100 mM phosphate buffer pH 7.4. Brains were frozen, 25 µm thick frontal sections were made on a Leica cryostat and processed as described previously (Goldschmidt et al. 2004, 2010).

### Data analysis

#### Photographic documentation

Digitized overviews from entire sections were obtained by scanning the sections at 2,000 dpi using an Agfa Duoscan T2000 XL. Details from sections were photographed with a Fuji Finepix S2 Pro digital camera on a Leica DMR microscope system. Photographs were arranged for illustrations using the Adobe Photoshop software on a PC. To quantify cerebral Tl^+^-uptake, 24-bit RGB color files from digitized sections were converted to unweighted 8-bit grayscale files and analyzed using ImageJ (NIH).

#### Quantification of relative Tl^+^-uptake rates

**Fig. 2:**
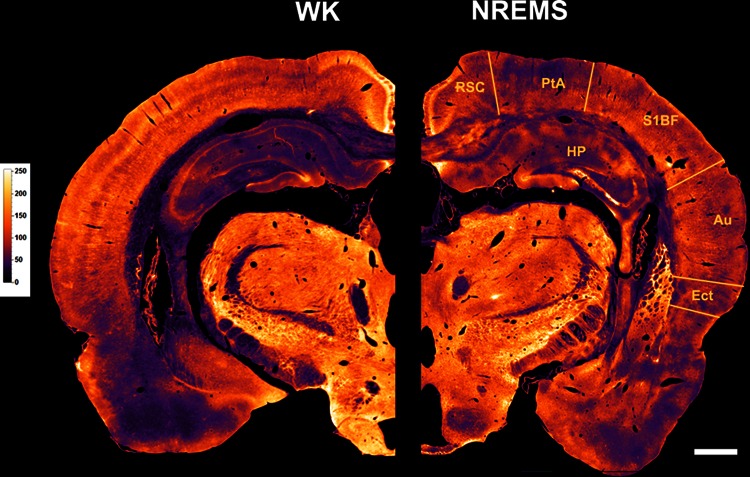
Overviews of Tl^+^-uptake in rat brains for WK and NREMS. Shown are frontal hemisections on the level of the dorsal hippocampus arranged as mirror images for WK (*left*) and NREMS (*right*). Images were transformed into 8-bit grayscale values and pseudocolored for better visualization, with *light colors* indicating a high level of Tl^+^-uptake and vice versa. For quantification of relative Tl^+^-uptake in cortical fields and the hippocampus, see Table 1. *Au* auditory cortex, *Ect* ectorhinal cortex, *HP* hippocampus, *PtA* parietal association cortex, *RSC* retrosplenial cortex, *S1BF* primary somatosensory cortex, barrel field. *Scale bar* is 1 mm

Relative Tl^+^-uptake in Table 1 was quantified by measuring mean gray level values in the respective brain regions of interest (ROIs) (Fig. 2). The grayscale was inverted in ImageJ, such that black corresponds to unstained pixels and white corresponds to pixels with the maximum staining intensity. ROI gray levels were then normalized by the mean gray value of the whole brain slice to obtain values for relative staining intensity of each ROI. This procedure is similar in rationale to the approach used by Ramm and Frost (1983) to quantify relative cerebral glucose consumption in sleeping rats. ROIs were determined based on the atlas of Paxinos and Watson (1998). Analyses were performed on 10–25 consecutive sections of one hemisphere for each animal. Statistical significance of group differences in relative Tl^+^-uptake was evaluated with a two-tailed Mann–Whitney *U* test.

**Table 1:** Relative Tl^+^-uptake in WK and NREMS

Structure	WK (*n* = 5)	NREMS (*n* = 6)	% Change	*p* Value
Auditory cortex	115.5 ± 2.2	115.0 ± 4.5	–	0.64
Ectorhinal cortex	82.4 ± 5.6	91.4 ± 5.5	+11	0.12
Hippocampus	80.2 ± 5.0	80.3 ± 3.3	–	0.65
Motor cortex	115.8 ± 4.4	110.8 ± 4.8	−4	0.41
Parietal association cortex	110.7 ± 4.4	100.5 ± 4.2	−9.2	0.12
Retrosplenial cortex	147.0 ± 8.2	138.2 ± 6.1	−6	0.52
Somatosensory cortex	117.2 ± 4.8	111.8 ± 4.3	−4.6	0.41
Visual cortex	117.4 ± 3.3	102.0 ± 6.0	−13	0.08
Whole neocortex	113.7 ± 3.2	107.0 ± 3.4	−6.3	0.17

Relative Tl^+^-uptake (% regional staining intensity/staining intensity of the whole slice) in neocortical areas and hippocampal formation for WK and NREMS. Values are mean ± SEM. Statistical significance of group differences was evaluated by a two-tailed Mann–Whitney *U* test. Note that % change in relative Tl^+^-uptake does not indicate changes in absolute metabolic rates between WK and NREMS, but only changes in the regional metabolic rate of the respective structure relative to the metabolic rate of the brain as a whole

**Fig. 3:**
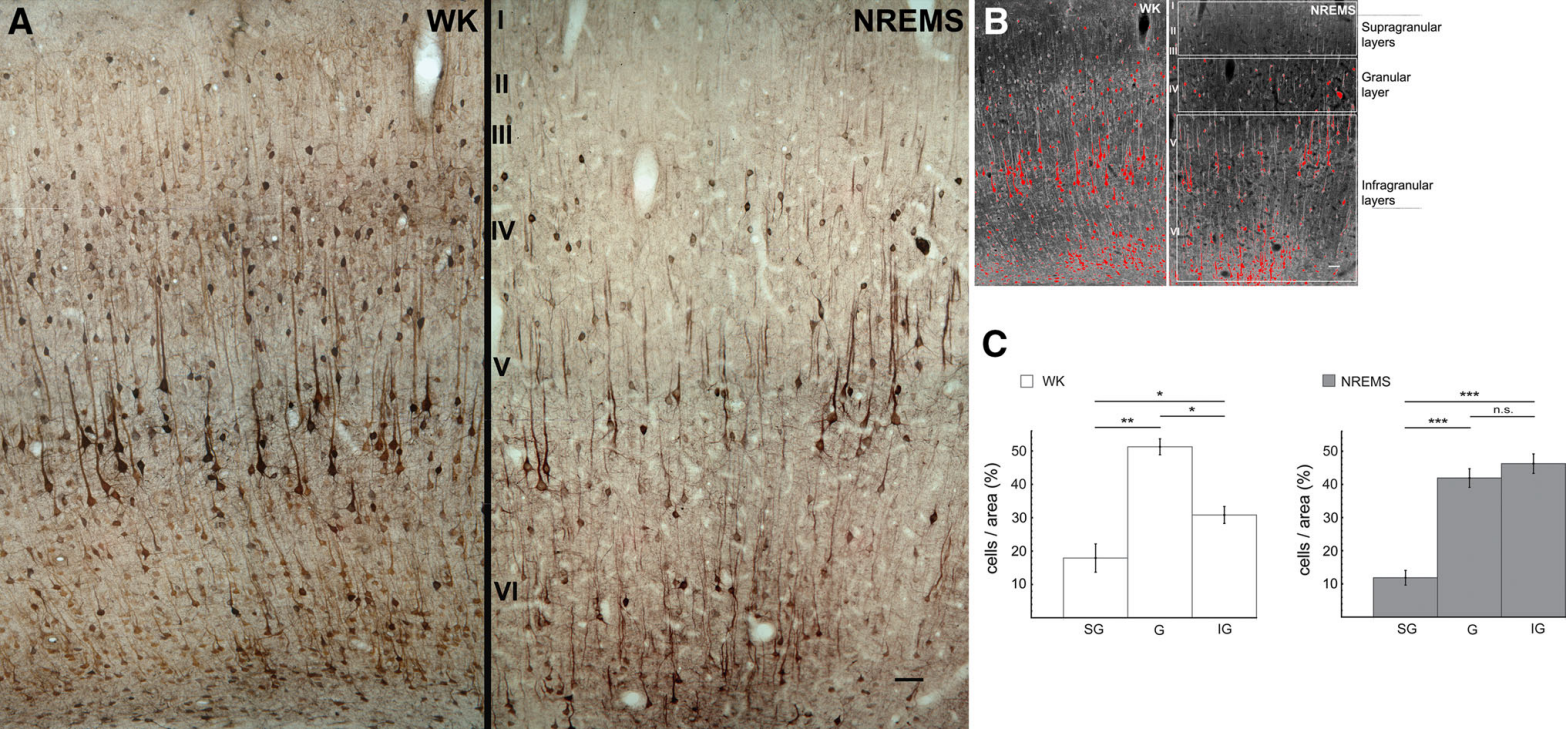
Laminar Tl^+^-uptake in primary auditory cortex (A1) for WK and NREMS. **a** Photomicrographs of A1 for WK (*left*) and NREMS (*right*). Under both conditions, a considerably higher fraction of intensely stained cells can be found in infragranular layers *V* and *VI* as compared to supragranular layers *I*, *II* and *III*. Note the reduced number of stained cells in granular layer *IV* of the NREMS animal. *Scale bar* is 50 µm. **b** Inverted grayscale images of the photomicrographs shown in **a**. Pixels were thresholded via ImageJ (NIH) and cells with *gray values* above threshold were counted using the built-in function “Analyze Particles” (see “Methods”). *White rectangles* indicate ROIs for supragranular, granular and infragranular layers, respectively. **c** Statistical analysis of laminar differences in cellular Tl^+^-uptake. Analyses were performed on 10 sections from different rostrocaudal levels of both hemispheres for each animal (*n* = 5 for WK, *n* = 6 for NREMS). *Percentage figures* indicate the ratio of suprathreshold cells in each ROI to the overall number of suprathreshold cells in the corresponding section. Data are mean ± SEM. Statistical significance of laminar differences was evaluated with a two-tailed Mann–Whitney *U* test (*p < 0.05; **p < 0.01; ***p < 0.005). *SG* supragranular layers, *G* granular layer, *IG* infragranular layers

**Fig. 4:**
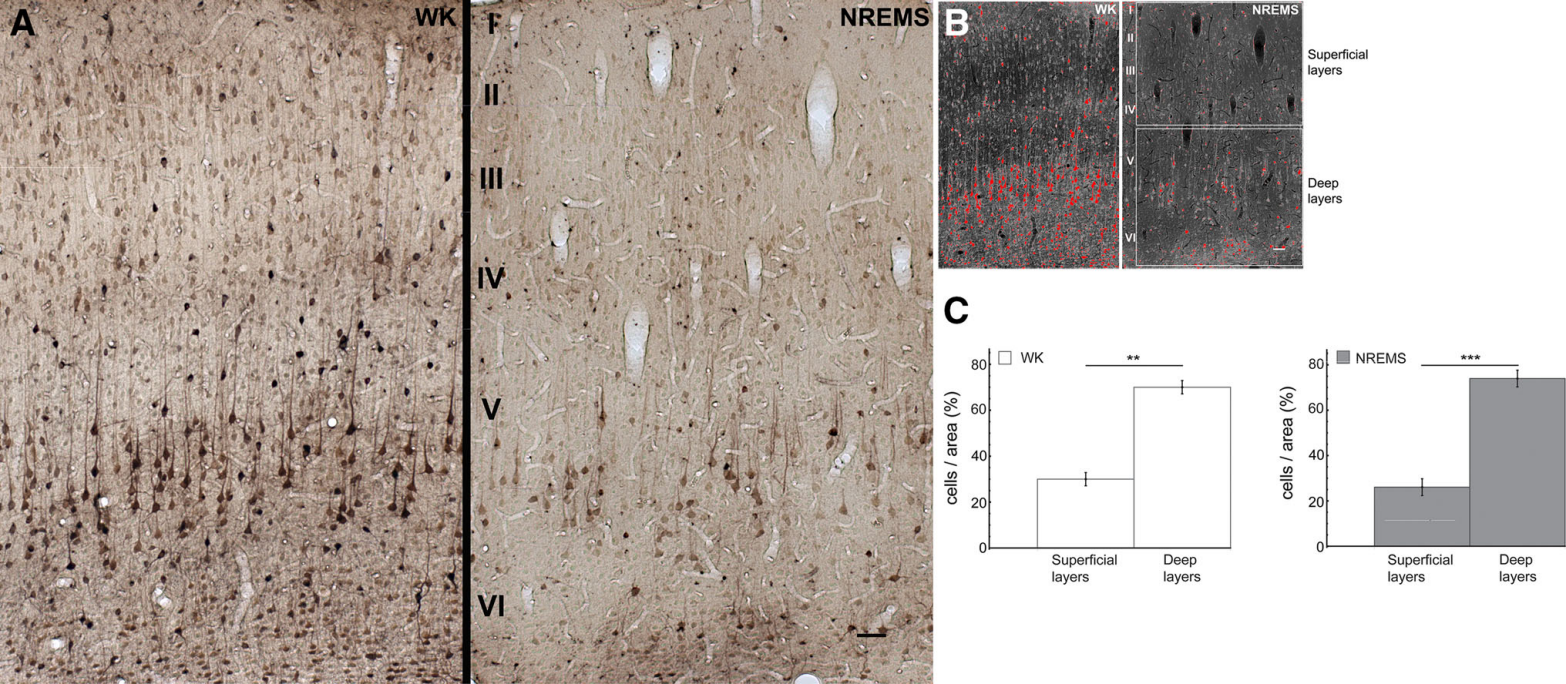
Laminar Tl^+^-uptake in parietal association cortex (PtA) for WK and NREMS. **a** Photomicrographs of PtA for WK (*left*) and NREMS (*right*). Under both conditions, a considerably higher fraction of intensely stained cells can be found in deep cortical layers as compared to superficial layers. *Scale bar* is 50 µm. **b** *Inverted gray scale* images of the photomicrographs shown in **a**. Pixels were thresholded as described for Fig. 3. *White rectangles* indicate ROIs for superficial and deep cortical layers. **c** Statistical analysis of laminar differences in cellular Tl^+^-uptake was performed as described for Fig. 3. Data are mean ± SEM (*n* = 5 for WK, *n* = 6 for NREMS)

**Fig. 5:**
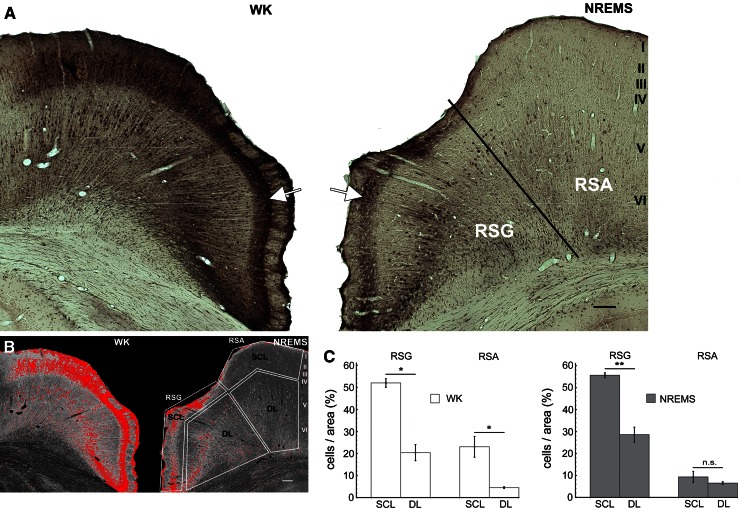
Laminar Tl^+^-uptake in retrosplenial association cortex (RSC) for WK and NREMS. **a** Photomicrographs of retrosplenial granular (RSG) and agranular cortex (RSA) for WK (*left*) and NREMS (*right*) arranged as mirror images. Note a distinct band of high Tl^+^-uptake in superficial layers of RSG for both WK and NREMS (*arrows*). Note also the comparatively low Tl^+^-uptake in superficial layers of RSA in the NREMS animal. *Scale bar* is 100 µm. **b** *Inverted grayscale* images of the photomicrographs shown in **a**. Pixels were thresholded as described for Figs. 3 and 4. *White polygons* indicate ROIs for superficial (SCL) and deep (DL) layers, respectively. **c** Statistical analysis of laminar differences in cellular Tl^+^-uptake was performed as described for Figs. 3 and 4. Data are mean ± SEM (*n* = 5 for WK, *n* = 6 for NREMS)

Cell counting in Figs. 3, 4 and 5 was performed in a similar fashion as described previously (Macharadze et al. 2012). In short, ROIs (10 sections from different rostrocaudal levels for each cortical area examined) were photographed at five times magnification, and colored photomicrographs were converted to unweighted, inverted grayscale images as described above. Images were normalized for contrast (0.4 % Saturated Pixels) and the scale was set to 0.454 pixels/µm. The mean gray value and the standard deviation of the mean (SD) were measured for each ROI and a threshold of 2 SD above the mean was defined. After setting the threshold for the whole ROI, sub-ROIs were defined based on the characteristic lamination patterns of each cortical area (see panel B in Figs. 3, 4, 5), as determined from adjacent Nissl-stained sections, using the chapter by Palomero-Gallagher and Zilles (2004) as a reference. Within each sub-ROI, cells with gray values above threshold were counted using the built-in function “Analyze Particles” (size: 23–300 corresponding to 111–1,450 µm^2^; circularity: 0.3–1). Data were then exported and further processed in Mathematica (Wolfram Research). Since our current Tl-AMG protocol does not allow for absolute quantification of Tl^+^-content, the number of cells/area in each sub-ROI was divided by the sum of the numbers of cells/area for all sub-ROIs, thus resulting in percentage figures (panel C in Figs. 3, 4, 5). Statistical significance of laminar differences was evaluated with a two-tailed Mann–Whitney *U* test.

#### Spatial spectral analysis

Potential occurrence of spatial periodicities in cortical Tl^+^-uptake during NREMS and WK was evaluated in unweighted, inverted grayscale images of scanned frontal Sects. (60–80 per animal) from different rostrocaudal levels (bregma: −1.3 to −6.3) in ImageJ (see panel A in Fig. 8 for illustration). Images were normalized for contrast as described above. ROIs encompassed the whole cortical mantle from the lateral edge of cingulate and retrosplenial areas to the ventral edge of insular and ectorhinal cortices. Within each ROI, the gray value distribution parallel to the cortical surface was obtained across all laminae using the “Segmented Line” tool (line width was adjusted for each ROI individually to account for variances in cortical thickness at different rostrocaudal levels) and the “Plot Profile” function. Data were exported and further processed in Mathematica.

Gray value profiles were filtered into a spatial frequency band of 0.25–15 cycles per millimeter (cycles/mm), and a Hamming window (length adjusted according to profile lengths) was applied to minimize spectral leakage. Next, signal power was standardized by subtracting the mean of squared values from each squared pixel value and dividing by the SD of the squared values. Finally, profiles were transformed from the spatial domain into the spatial frequency domain using discrete Fourier transform (DFT), and power spectral densities (PSDs) were estimated. No correction was made for tissue shrinkage in assessing spatial bandwidth.

To control for circadian time as a potential confounding factor, some of the above analyses were performed on additional data from rats which were awake in the morning (9–11 am). In this group (*n* = 4), WK was not monitored by polygraphic recordings, but instead was verified by visual inspection. Moreover, as this circadian control group was recruited from a database of rats which served as the control group for a study on the visual system, only the caudal portion of the cerebrum (Bregma −5.60 to −6.30) was available for analysis. Therefore, comparisons between the early (WK_am_) and the late (WK_pm_) WK group were made only on equivalent rostrocaudal levels to account for potential rostrocaudal differences in tracer uptake.

## Results

### Relative Tl^+^-uptake in cortical regions does not significantly differ between NREMS and WK

It is relatively well established that the metabolic rate of the brain as a whole decreases from WK to NREMS, based on human and animal studies using glucose consumption (Kennedy et al. 1982; Nofzinger et al. 2002) and brain perfusion (see Maquet 2000 for a comprehensive review) as an index. However, some controversy abounds regarding the degree to which different cortical areas change their metabolic rate in proportion to the observed global decline in brain metabolism. While human PET-studies of brain perfusion concordantly report decreases in regional cerebral blood flow throughout the cortex (Braun et al. 1997; Hofle et al. 1997; Maquet et al. 1997; Andersson et al. 1998; Kajimura et al. 1999), Nofzinger et al. (2002) observed relative increases in regional glucose consumption in various structures, including primary sensorimotor cortex and associational cortices. Moreover, differences in the degree of “deactivation” between frontoparietal association areas and unimodal sensory areas have been pointed out (Braun et al. 1997; Kajimura et al. 1999; Nofzinger et al. 2002), suggesting that cortical regions involved in higher order processing might be disproportionately affected by the restorative or homeostatic influence of NREMS.

To test the extent to which functionally separable fields of the rat cortex are differentially affected by the metabolic changes accompanying NREMS, we compared regional Tl^+^-uptake relative to global Tl^+^-uptake in various cortical fields and the hippocampus across the vigilance states (Fig. 2). Of the eight regions examined, none showed a statistically significant change in relative Tl^+^-uptake from WK to NREMS, as indicated by relative staining intensities in Table 1. Visual cortical areas showed the strongest trend towards a decrease in relative Tl^+^-uptake (−13 %), whereas the ectorhinal cortex was the only cortical region where a noticeable trend towards an increase in relative Tl^+^-uptake (+11 %) from WK to NREMS was observed. Of note, it should be emphasized that the regional changes in Tl^+^-uptake rates listed in Table 1 do not reflect absolute changes in Tl^+^-uptake, but only relative changes in comparison to global Tl^+^-uptake within each vigilance state.

### Laminar Tl^+^-uptake in NREMS and WK

The laminar structure of the mammalian neocortex is a defining means of functional cortical circuitry, with principal cells in each layer possessing a unique profile with respect to their afferent and efferent connections as well as to their morphological and physiological characteristics (Douglas and Martin 2004; Bannister 2005). How the laminar activity profiles of active desynchronized states such as WK are affected by global network state in relation to synchronized states like NREMS is currently an area of intense research (Poulet and Petersen 2008; Sakata and Harris 2009; Wanger et al. 2013). To gain insight into the functional laminar circuitry of NREMS, we quantified laminar Tl^+^-uptake patterns for a representative unimodal area (A1), a representative multimodal area (PtA) and a limbic association area (RSC).

#### Primary auditory cortex (A1)

While on the macroscopic level, relative Tl^+^-uptake in auditory cortex as compared to global Tl^+^-uptake did not differ between NREMS and WK (Table 1), notable differences in laminar Tl^+^-uptake were observed (Fig. 3). In the WK group, the number of intensely stained cells was highest in granular layer IV of A1 [Fig. 3c, mean_SG_ = 17.9 % ± 4.2, mean_G_ = 51.3 % ± 2.4 and mean_IG_ = 30.8 % ± 2.5 (±SEM)]. For the NREMS group in contrast, the peak of the laminar cell count was shifted towards infragranular layers V and VI [Fig. 3c, mean_SG_ = 11.8 % ± 2.2, mean_G_ = 41.9 % ± 2.8 and mean_IG_ = 46.2 % ± 2.9 (±SEM)]. In both WK and NREMS groups, cell counts in supragranular layers were significantly lower as compared to counts in both granular and infragranular layers (Fig. 3c).

In the circadian control group (WK_am_), a relative increase in infragranular cell counts was observed when compared to the WK (WK_pm_) group (Suppl. Figure 2).

#### Parietal association cortex (PtA)

PtA represents a hierarchically higher cortical field where responses from different sensory modalities are integrated (Toldi et al. 1986; Lippert et al. 2013). As granular layer IV in rat association areas is typically very sparse (Miller and Vogt 1984; Lippert et al. 2013), analysis of laminar Tl^+^-uptake was restricted to superficial and deep layers (Fig. 4b), with superficial ROIs encompassing sparse layer IV as determined by neighboring Nissl sections (Suppl. Figure 3).

As for A1, Tl^+^-uptake in NREMS was generally most pronounced in deeper layers of PtA (Fig. 4). In particular, numbers of intensely stained cells differed significantly between deep layers (DL) and superficial layers (SCL) for both WK (Fig. 4c, mean_SCL_ = 30 % ± 2.9 and mean_DL_ = 70 % ± 2.9 (±SEM), *p* < 0.01) and NREMS (Fig. 4c, mean_SCL_ = 26 % ± 3.7 and mean_DL_ = 74 % ± 3.7 (±SEM), *p* < 0.005).

#### Retrosplenial association cortex (RSC)

RSC is one of the largest cortical regions in the rat (Vogt and Peters 1981; van Groen and Wyss 1990) and is known to show high levels of spontaneous activity in both WK and NREMS. This is reflected in the relative Tl^+^-uptake rates of the RSC (Table 1). Further, the RSC has been noted as an important hub of the rat default-mode network (Upadhyay et al. 2011) with a central role for memory, cognition and navigation (Vogt and Vogt 2004; Vann et al. 2009). Since the rat RSC is usually subdivided into a granular (RSG) and a dysgranular or agranular (RSA) part based on its cytoarchitecture and connectivity (van Groen and Wyss 1990), quantitative analysis of laminar Tl^+^-uptake was performed separately for RSG and RSA (Fig. 5).

Notably, and in contrast to A1 and PtA, cell counts in superficial layers (SCL) of RSG were significantly higher as compared to deep layers (DL) across vigilance groups [Fig. 5c, WK: mean_SCL_ = 52 % ± 2 and mean_DL_ = 20.4 % ± 3.7 (±SEM), *p* < 0.05; NREMS: mean_SCL_ = 55.6 % ± 1.1 and mean_DL_ = 28.6 % ± 3.4 (±SEM), *p* < 0.01]. In contrast, for RSA, cell counts in superficial layers were only significantly higher as compared to deep layers for WK [Fig. 5c, mean_SCL_ = 23 % ± 4.7 and mean_DL_ = 4.5 % ± 0.4 (±SEM), *p* < 0.05), whereas no difference was found for NREMS (Fig. 5c, mean_SCL_ = 9.3 % ± 2.6and mean_DL_ = 6.5 % ± 0.66 (±SEM), *p* = 0.58].

Of note, distinct labeling of dendritic bundles was observed frequently in layer I of RSG for both WK and NREMS (Suppl. Figure 4).

### Cortical Tl^+^-uptake is not spatially homogeneous

Accumulating evidence indicates that NREMS is regulated on a regional level in the cortex in a use-dependent fashion (Kattler et al. 1994; Huber et al. 2004; Rector et al. 2005; Vyazovskiy et al. 2011), and it has been suggested that sleep is a property of individual cortical assemblies or cortical columns (Krueger et al. 2008). Hence, if NREMS is indeed regulated on a local scale, such that during whole animal sleep some neuronal assemblies remain in a wake-like state and vice versa (Krueger et al. 2008), then this should be reflected in cortical Tl^+^-uptake patterns of spontaneous NREMS and/or WK episodes.

**Fig. 6:**
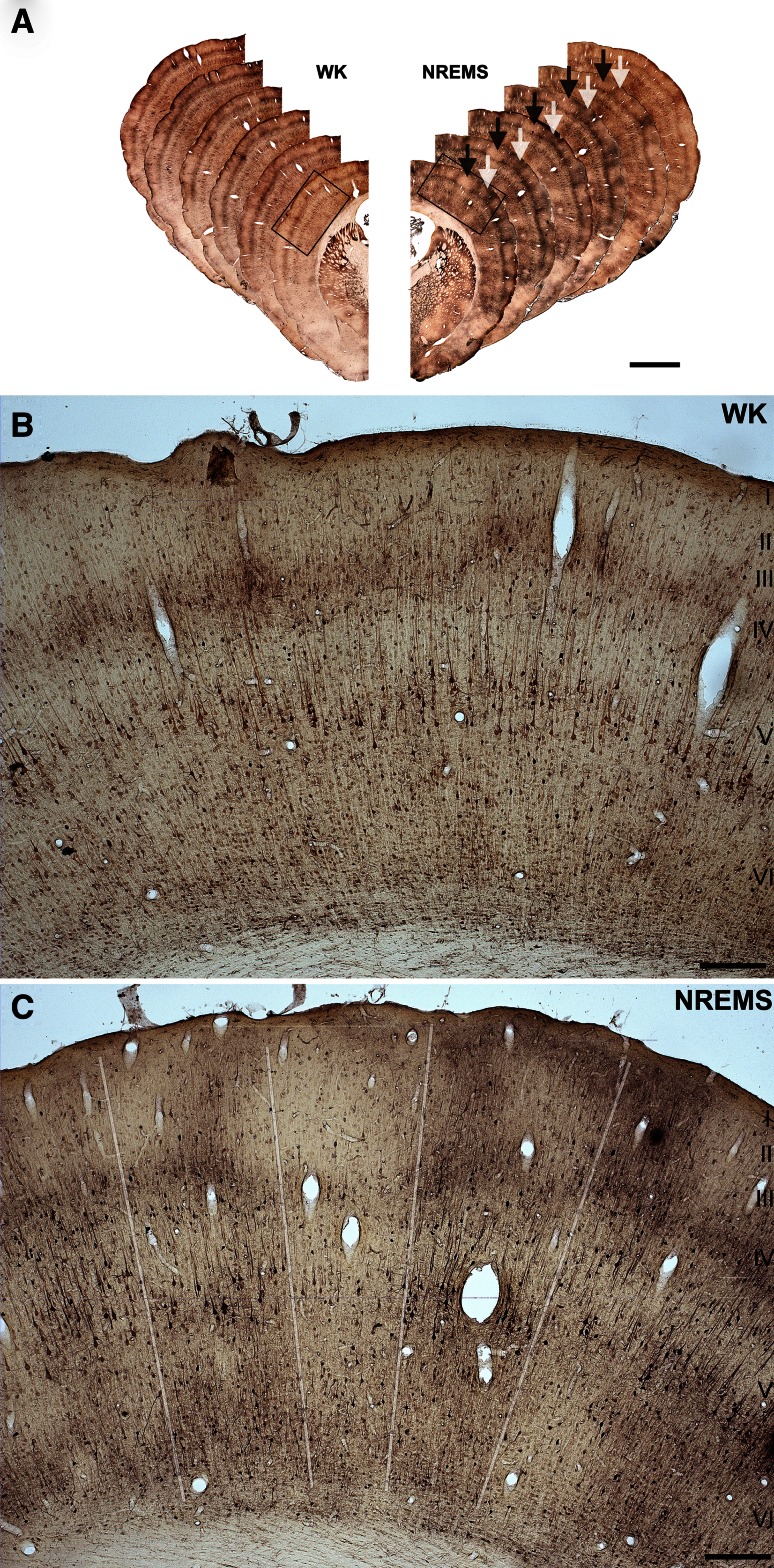
Highly salient columnar Tl^+^-uptake patterns in NREMS. **a** Shown are series of frontal hemisections on the level of the barrel field (S1BF) arranged as mirror images for WK (*left*) and NREMS (*right*) from rostral (*front*) to caudal (*back*). Note columns of high and low Tl^+^-uptake in sections of the NREMS animal as indicated by *white and black arrows*, respectively. *Black rectangles* indicate the position of the details shown in **b** and **c**. **b**, **c** Photomicrographs of S1BF as indicated in *panel A* for WK (**b**) and NREMS (**c**). Note pronounced columnar uptake patterns in **c** as indicated by semitransparent* white lines*. In contrast, Tl^+^-uptake in **b** appears to be more homogeneous parallel to the cortical surface. *Scale bar* is 2 mm in **a** and 250 µm in **b** and **c**

**Fig. 7:**
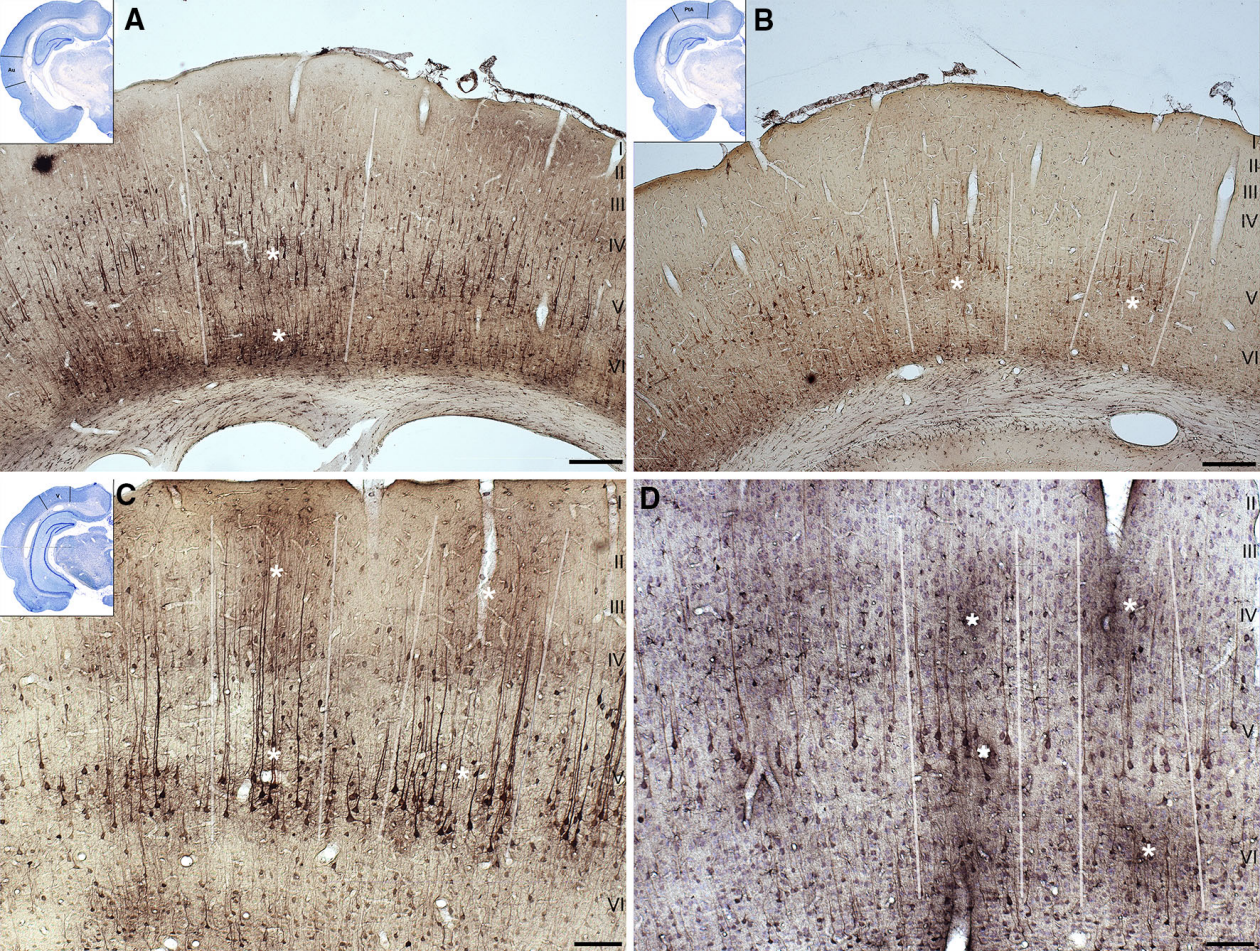
Columnar Tl^+^-uptake patterns are found in various unimodal and multimodal areas. Shown are photomicrographs of auditory cortex (**a**), parietal association cortex (**b**) and visual cortex (**c**, **d**) from NREMS animals. *Insets* in **a**, **b**, **c** indicate the rostrocaudal position of the photomicrographs in frontal overviews of neighboring Nissl sections. The section in **d** was doublestained for Tl^+^ and Nissl. Columnar uptake patterns in **a**–**d** are indicated by semitransparent *white lines*. Note that Tl^+^-uptake clusters (*white asterisks*) are most prominent in infragranular layers (**a**–**d**), but sometimes extend to or include supragranular (**c**) and granular layers (**d**). Note also that columnar Tl^+^-uptake is not related to local inhomogeneities in cell density as is shown in **d**. *Scale bar* is 250 µm in **a** and **b** and 100 µm in **c** and **d**

In fact, clearly delineable clusters of Tl^+^-uptake were frequently observed in unimodal and multimodal areas throughout the neocortex across vigilance groups, but were more salient in NREMS animals (Figs. 6, 7). Tl^+^-uptake within a cluster often extended to all cortical layers (Figs. 6c, 7c, Suppl. Figure 5), but, in accordance with the laminar uptake patterns described above, was most prominent in infragranular layers (Fig. 7a, b) for both intensely and weakly labeled columns (Suppl. Figure 6). Furthermore, double staining with Nissl confirmed that columnar uptake patterns were not due to differences in cell densities (Fig. 7d). Interestingly, salient columnar Tl^+^-uptake patterns were also observed in the CA1 region of the hippocampus of some NREMS animals (Suppl. Figure 7), and, though less frequently, in the cerebellar cortex of both NREMS and WK animals (Suppl. Figure 8).

**Fig. 8:**
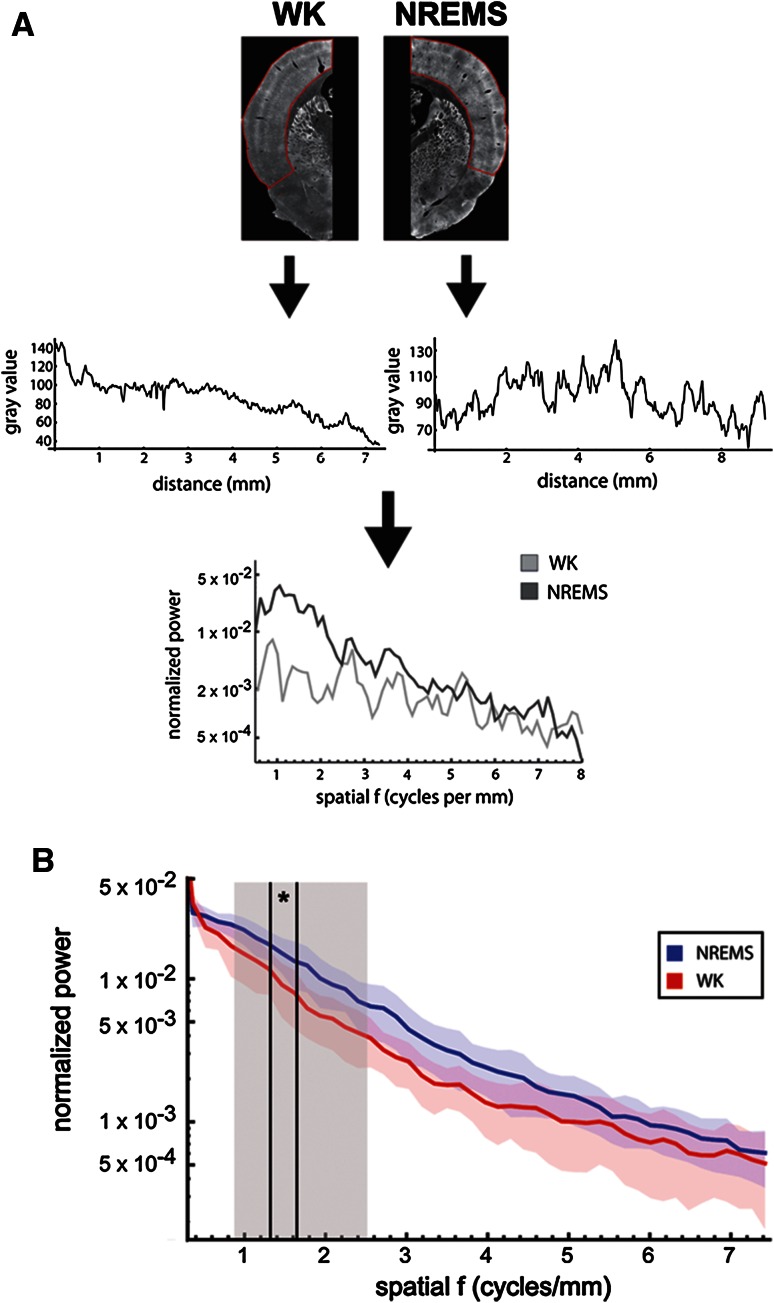
Quantification of spatial periodicities in cortical Tl^+^-uptake. **a** Analyses were performed on inverted grayscale images of scanned frontal Sects. (60–80 per animal) for both WK and NREMS. ROIs included the whole cortical mantle from the lateral edge of cingulate and retrosplenial areas to the ventral edge of insular and ectorhinal cortices (*red contours*, *top*). Within each ROI, the *gray value* distribution parallel to the cortical surface was obtained across all laminae (*middle*) and processed to obtain power spectral densities (PSDs) for the spatial frequency (*bottom*). **b** Grand average of normalized PSD plots obtained as described above (semi-logarithmic scale). Shown are means (*thick lines*) ± standard deviations of the mean (*blue and red shaded areas*) for WK (*red*, *n* = 5) and NREMS (*blue*, *n* = 6). Note the absence of clear spectral peaks in normalized PSD plots of both vigilance groups, indicating that periodicities in cortical Tl^+^-uptake were not strongly biased towards any spatial wavelength. The *gray shaded area* (0.83–2.5 cycles/mm) indicates the spatial frequency bin that corresponds to reported diameters for cortical macrocolumns (200–600 µm). Mean PSD values for NREMS and WK were found to differ significantly within that frequency bin (*t* test, *p* < 0.005). Post-hoc analysis of individual PSD mean value pairs revealed that normalized power was significantly higher in NREMS as compared to WK for a spatial frequency bin of 1.33–1.64 cycles/mm (indicated by *vertical black lines*, two-tailed Mann–Whitney *U* test, *p < 0.05)

In order to test for spatial periodicities in cortical Tl^+^-uptake, we computed the power spectrum of the normalized spatial profiles in the tangential plane (parallel to the cortical surface, Fig. 8a). In both vigilance groups, power spectral densities (PSDs) followed a 1/*f* distribution, but no clear spectral peaks were observed (Fig. 8b). The absence of spectral peaks indicates that periodicities in cortical Tl^+^-uptake were not strongly biased towards any spatial wavelength, but that instead, spatial clustering of Tl^+^-uptake did occur on various spatial scales and was often aperiodic or irregular. In other words, the spatial up and down of Tl^+^-uptake did not manifest in form of regularly alternating clusters of a fixed diameter, but instead, functional clusters exhibited variable diameters and irregular spacing.

Spatial spectral analysis also confirmed the higher saliency of Tl^+^-uptake clusters in NREMS as compared to WK animals. PSD values between a spatial frequency range of 0.5–7.5 cycles/mm (corresponding to half-cycle lengths or cluster diameters of 65 µm–1 mm) were generally biased towards higher values in NREMS (Fig. 8b). To test for potential effects of vigilance state on periodicities in Tl^+^-uptake at the scale of cortical columns, we performed a *t* test on the normalized mean PSD values for NREMS and WK within a spatial frequency band from 0.83 to 2.5 cycles/mm [corresponding to columnar diameters of 200 µm up to 600 µm (Mountcastle 1997; Defelipe et al. 2012)]. The difference in PSD means between the two vigilance groups within the “columnar” band proved to be highly significant (mean_WK_ = 8.7 × 10^−3^ ± 1.3 × 10^−3^ and mean_NREMS_ = 1.4 × 10^−2^ ± 1.7 × 10^−3^ (±SEM), *p* < 0.005). To determine which spatial frequency bins were affected in particular, we performed a post hoc comparison of normalized PSD mean values for individual sample points within the spatial frequency band of 0.83–2.5 cycles/mm. For a spatial frequency bin of 1.33–1.64 cycles/mm, individual PSD values differed significantly between groups (two-tailed Mann–Whitney *U* test, *p* < 0.05), whereas for bins of 1.8–2.12 and 2.27–2.5 cycles/mm, differences in PSD values were close to significance threshold (*p* = 0.056 for each bin). Comparison of individual PSD values between the WK group (WK_pm_) and the circadian control group (WK_am_) did not reveal any statistically significant differences within the spatial frequency band of 0.83–2.5 cycles/mm (Suppl. Fig. 9).

These results show that on the scale of anatomically defined cortical macrocolumns, periodicities in cortical Tl^+^-uptake show a small but significant bias towards higher contrast in NREMS as compared to WK.

## Discussion

We used TlAMG to map cortical activity patterns during spontaneous episodes of NREMS in freely moving, unrestrained rats. Our main findings are (1) regional Tl^+^-uptake rates relative to global uptake do not differ between WK and NREMS for cortical fields of various modality and hierarchy; (2) laminar Tl^+^-uptake in NREMS is generally most pronounced in infragranular layers V and VI; and (3) distinct columnar Tl^+^-uptake patterns were observed in NREMS across the cortical mantle.

Our data show that cortical activity patterns in NREMS are not spatially homogenous, but rather are as complex, if not more complex, than in WK. Hence, our study strongly argues against a spatially ubiquitous reduction in cortical metabolic rate from WK to NREMS, but in turn provides support for the view of NREMS as a local phenomenon, emphasizing the predominant role of deep layers.

### Cortical metabolism in NREMS

Human and animal studies looking at cerebral metabolism during the deep stages of NREMS concordantly report global decreases in either glucose (Buchsbaum et al. 1989; Kennedy et al. 1982; Maquet et al. 1990; Nofzinger et al. 2002; Ramm and Frost 1986) or oxygen (Madsen et al. 1991) consumption by 25–40 % as compared to WK. In addition, human studies on brain perfusion during NREMS in unison report decreases in the rates of regional cerebral blood flow from WK to deep NREMS, in cortical as well as subcortical areas (Braun et al. 1997; Hofle et al. 1997; Maquet et al. 1997; Andersson et al. 1998; Kajimura et al. 1999). These global reductions in cerebral metabolic rate are also reflected in Tl^+^-uptake rates and are particularly notable in the cortex (compare the overall staining intensities WK vs. NREMS of the cortical mantle in Fig. 2 and of individual cortical areas in Figs. 3, 4, 5). However, as the current TlAMG-protocol allows only for semi-quantitative comparisons between different animals, which are based on normalizing local Tl^+^-uptake rates to overall Tl^+^-uptake rates of the corresponding section, we make no explicit statements with respect to absolute values of Tl^+^-uptake in WK and NREMS. Instead, we confine our analysis to comparing regional Tl^+^-uptake rates relative to global Tl^+^-uptake within individual cortical fields (cf. Ramm and Frost 1983).

Human PET-studies have shown that NREMS-related decreases in cortical metabolic rate are more pronounced in frontoparietal association cortices than in unimodal sensory cortices (Braun et al. 1997; Kajimura et al. 1999; Nofzinger et al. 2002). It has been suggested that these disproportional decreases in local metabolism might reflect a use-dependent bias in the degree to which neural populations in cortical areas of different hierarchical levels are affected by the synchronous slow sleep oscillations of deep NREMS (Maquet 2000). Notably, our data indicate no marked differences between unimodal and multimodal/associational areas in the degree of change in relative metabolic rate from WK to NREMS (Table 1). Both unimodal sensorimotor areas as well as multimodal and associational areas showed non-significant trends towards decreases in relative Tl^+^-uptake rates for NREMS between 4 and 13 %, with the exception of auditory areas (no change) and the ectorhinal cortex (+11 %). We again want to emphasize that the absence of statistically significant changes in relative Tl^+^-uptake from WK to NREMS does not indicate that absolute metabolic rates are of similar magnitude in the two conditions, but, rather, that the metabolic rate in cortical fields relative to the metabolic rate of the brain as a whole does not markedly differ between WK and NREMS. In other words, none of the cortical areas examined, including the hippocampus, shows a significantly stronger decline in its activity from WK to NREMS than the rest of the brain.

We thus conclude that, at least in the rat neocortex, hierarchy of processing does not predict the degree of change in relative metabolic rate for NREMS.

### Predominance of infragranular layers in NREMS

The observed predominance of infragranular activation in both unimodal and multimodal areas (Figs. 3, 4), though not explicitly reported by any metabolic study so far, does not come as a surprise in light of recent physiological data. For instance, during deep NREMS and under the influence of most anesthetics, cortical neurons continuously and synchronously cycle between depolarized up states corresponding to generalized network activity and hyperpolarized down states of network silence, which is reflected in depth local field potential and electrocorticogram recordings as a slow oscillation (Steriade et al. 1993, 2001). There is now accumulating evidence indicating that layer V networks play a leading role in the initiation, sustainment and synchronization of cortical up states (Sanchez-Vives and McCormick 2000; Sakata and Harris 2009; Chauvette et al. 2010; Beltramo et al. 2013; Stroh et al. 2013) and slow-wave propagation (Luczak et al. 2007; Wester and Contreras 2012; Stroh et al. 2013). In particular, layer V pyramidal cells have been shown to be causally involved in the regulation of up and down state dynamics in anesthetized mice (Beltramo et al. 2013). Moreover, multi-unit activity in the auditory cortex of urethane anesthetized rats is densely distributed in layer V networks, while being sparse and spatially localized in supragranular layers (Sakata and Harris 2009).

However, as most of these studies were conducted either under anesthesia or in slices, our study is the first to confirm a dominant recruitment of infragranular networks for natural NREMS on a larger cortical scale, using unbiased metabolic mapping of activity on the cellular level. As this dominance of infragranular or deep layers over supragranular layers also extends to WK, though to a lesser degree, our data unambiguously demonstrate that, at least in the rat, the bulk of metabolic processing takes place in the cortical output layers V and VI across cortical states. This complements spike data showing that neural firing is much less sparse in deep cortical layers as compared to superficial layers across various anesthetized and awake preparations (Barth and Poulet 2012) and emphasizes the prevalence of deep layers in the cortical microcircuitry.

In addition, the finding of a comparatively reduced granular layer IV metabolism in NREMS (Fig. 3) confirms earlier studies using 2-DG as a metabolic marker (Kennedy et al. 1982; Ramm and Frost 1983, 1986) and seems to be readily explained by reduced thalamocortical input in NREMS. In primary sensory cortex, layer IV is the major recipient of afferent fibers from specific thalamic nuclei which exert a strong driving force on the cortex (Douglas and Martin 2004; Bannister 2005). During NREMS, thalamocortical sensory transmission is considerably diminished by the inhibitory influence of the reticular thalamic nucleus (Steriade 2000; Jones 2002), which consequently might result in diminished processing of sensory-evoked thalamic input within cortical layer IV, hence reducing the metabolic burden on that layer. However, in addition to these state-dependent changes in thalamic activity, local use-dependent processes may influence thalamocortical sensory transmission in NREMS (Rector 2009), and may thus contribute to the reduced metabolic load in cortical layer IV.

A striking exception to the “rule” of deep layer predominance is posed by the laminar activity distribution in the retrosplenial cortex (RSC) which stands out from the rest of the neocortex by its comparably extensive degree of superficial layer activation (Fig. 5). Given that the RSC is a main hub in the rat default mode network (Upadhyay et al. 2011) with exceptionally high resting metabolism (Table 1), this reversal in the laminar activity profile potentially reflects the heavy degree of subcortical and cortical innervation the RSC receives compared to other cortical areas (Vann et al. 2009), most of which is targeted at superficial layers including sparse layer IV (Vogt and Vogt 2004).

Of particular note is the pronounced relative decline in activity within superficial layers of the RSA subregion from WK to NREMS (Fig. 5c). For RSG, in contrast, no such decline in superficial layer activity was observed (Fig. 5c). This subregional difference in the state-dependency of the RSC can, in all likelihood, be attributed to the different weighting in external (RSA) vs. internal (RSG) information processing within the two subregions. For instance, RSA but not RSG receives prominent visual input from both the laterodorsal thalamic nucleus and the visual cortex (Thompson and Robertson 1987; van Groen and Wyss 1992) and is supposed to be an important area for visuospatial information processing based on external sensory information (Pothuizen et al. 2009). RSG, in contrast, connects heavily with other limbic structures like the anterior thalamic nuclei and the subiculum (van Groen and Wyss 1990) and is thought of to be more involved with spatial memory processing based on internal cues (Pothuizen et al. 2009). Hence, we interpret this subregion-selective decrease in superficial layer activation as indicative of a disproportional decline in external vs. internal information transfer to the RSC during NREMS.

### Columnar activity patterns in NREMS

Our finding that cortical activity patterns are often marked by visible clusters or columns of higher and lower Tl^+^-uptake is well in line with electrophysiological and imaging data showing that NREMS is regulated in a spatially non-uniform fashion (Pigarev et al. 1997; Huber et al. 2004, 2006; Rector et al. 2005; Nir et al. 2011; Vyazovskiy et al. 2011). The “as-if-stimulated” appearance of large parts of the neocortex in NREMS (Figs. 6, 7) shows that, at least on a time scale of 5 min, some neuronal groups in the rat cortex are more metabolically active than others, implying local differences in both postsynaptic input and action potential firing of cortical assemblies. Furthermore, spatial spectral analysis of these activity clusters revealed that, for a narrow spatial frequency band of 1.33–1.64 cycles/mm (corresponding to half-cycle lengths or cluster diameters of approximately 300–400 µm), the magnitude of clustering was, on average, significantly stronger in NREMS as compared to WK (Fig. 8b). These corresponding diameters lie well within the range of sizes reported for cortical macrocolumns (Mountcastle 1997; Defelipe et al. 2012), which indicates that spatial clustering has its strongest sleep-dependent manifestation on the scale of anatomically defined columns. In other words, alternations between active and less active neuronal assemblies were of significantly higher contrast in NREMS as compared to WK, particularly for assemblies the size of cortical columns.

This observation supports the prediction that during NREMS, some cortical columns are in a sleep-like state, i.e. show reduced levels of metabolism, while other columns can still exist in an awake-like state and show comparatively high levels of metabolism (Krueger and Obál 1993; Krueger et al. 2008). Underpinning this interpretation is the fact that functional clustering was also observed in awake animals, but was less abundant and less well-defined in this vigilance group. It should be noted that when referring to neuronal assemblies as “awake” or “asleep,” respectively, we do so based on the assumed firing patterns and membrane potential dynamics in these assemblies (Destexhe et al. 2007; Krueger et al. 2013) and we explicitly do not rule out different local intensities in slow-wave activity as a potential underlying mechanism. For instance, as slow oscillation down states can occur as spatially localized phenomena (Sirota and Buzsáki 2005; Nir et al. 2011; Vyazovskiy et al. 2011), columnar Tl^+^-uptake patterns could be explained by local differences in the density of down and up states, respectively, with local down states being, on average, more prevalent in columns where Tl^+^-uptake was comparatively low. Underpinning this interpretation is the fact that even in “awake” columns, laminar Tl^+^-uptake patterns show the typical signature of NREMS, lacking the prominent granular layer activation of global WK (Suppl. Figure 6).

Whatever the underlying biological mechanisms may be, we emphasize that the spatial cortical activity pattern in NREMS is not to be mistaken for a ubiquitous “on and off” pattern of alternating functional columns of similar diameter. Rather, functional clusters were of variable diameter and occurred with no preferred spatial frequency, a fact that is reflected in the absence of spectral peaks in the spatial power spectrum (Fig. 8b).

Columnar activity patterns have not been reported in the only other metabolic study of rat NREMS where 2-DG was used as a tracer (Ramm and Frost 1983). The reason for this discrepancy may lie in the longer integration period required in the former study. There, circulating blood 2-DG levels had to be correlated with the time the animal spent in different vigilance states during the 45-min postinjection period. This approach requires functional clusters to remain stable beyond the 5-min time frame investigated in our study, which is unlikely to be the case during unstable and short lasting states like rat NREMS. Rather, functional clusters of high and low activity neurons probably shift or change during state transitions such as NREMS to WK or NREMS to REMS, which repeatedly occur during an integration period of 45 min. This interpretation is supported by two other 2-DG studies where, in fact, distinctive columnar patterns of 2-DG consumption were observed in rat neocortex during more stable states of drowsiness as induced by systemic applications of irreversible alpha-adrenergic blockers (Savaki et al. 1982) and subanesthetic doses of Ketamine (Hammer and Herkenham 1983).

However, to which degree the spatial metabolic pattern reported here actually reflects use-dependent differences in sleep intensity (Tononi and Cirelli 2006; Krueger et al. 2008; Rattenborg et al. 2009) or actually represents a metabolic correlate of localized replay/reactivation of activity patterns acquired during prior WK (Diekelmann and Born 2010; O’Neill et al. 2010) cannot be addressed in this study.

In conclusion, our study is the first to use an unbiased, non-invasive approach to map cortical activity on a cellular level during discrete (5 min) spontaneous NREMS episodes in the rat. Our findings demonstrate that cortical activity in NREMS is spatially diverse and shows a strong bias towards neuronal assemblies located in deep layers.

## Electronic Supplementary Material

Below is the link to the electronic supplementary material.

Supplementary material 1 (PDF 995 kb)
