# Supplementary material for: Spatial patterns of neuronal activity in rat cerebral cortex during non-rapid eye movement sleep
Source: Brain Struct Funct. 2014 Aug 13;220(6):3469–84. doi: 10.1007/s00429-014-0867-9 (PMC4575691; doi:10.1007/s00429-014-0867-9)
Supplement: Supplementary file 1 — Supplementary material 1 (PDF 995 kb) [file 429_2014_867_MOESM1_ESM.pdf]

Spatial patterns of neuronal activity in rat cerebral cortex during non-rapid eye movement sleep (Wanger et al.): Supplementary data

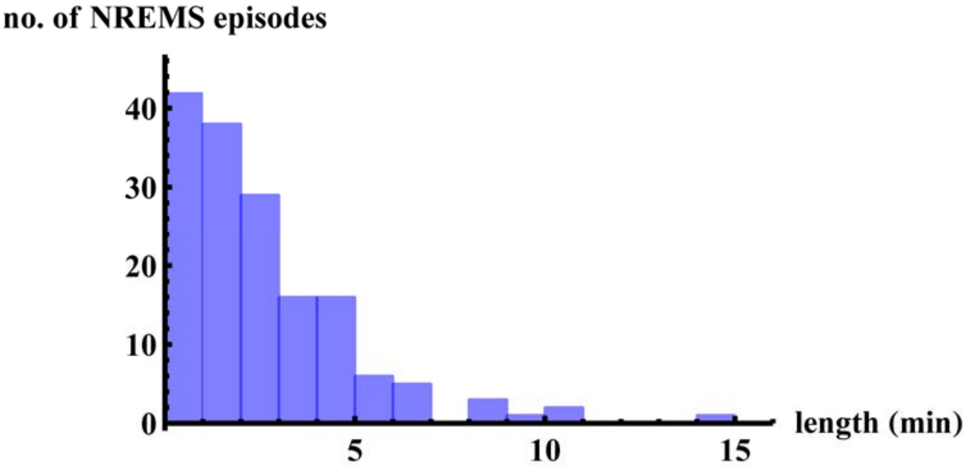


*Supplementary figure 1.* Histogram showing the durations of discrete NREMS episodes (pooled data from three animals, 4 - 5 h of polygraphic recordings per animal). The majority of episodes (~80%) are shorter than 4 min.

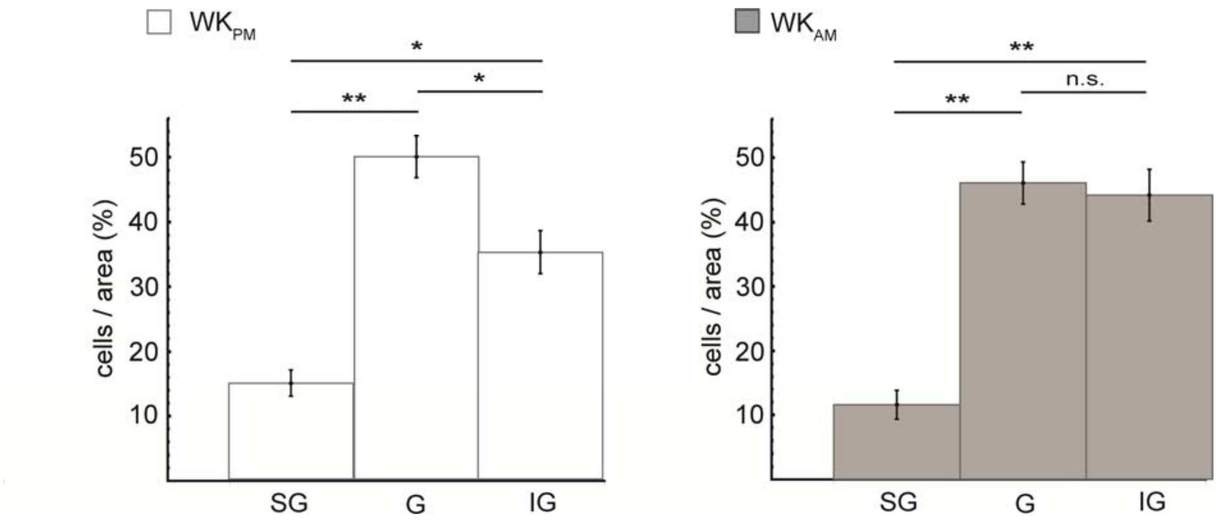


*Supplementary figure 2.* Laminar $Tl^+$-uptake in primary auditory cortex (caudal portion, Bregma ~ -5.80) for wakefulness in the evening (between 6pm and 8pm, $WK_{pm}$) and in the morning (between 9am and 11am, $WK_{am}$). Analyses were performed on 10 sections from both hemispheres for each animal (n = 5 for $WK_{pm}$, n = 4 for $WK_{am}$). Percentage figures indicate the ratio of suprathreshold cells in each laminar ROI to the overall number of suprathreshold cells in the corresponding section. Data are means ± SEM. Statistical significance of laminar differences was evaluated with a two-tailed Mann-Whitney U-test. SG: supragranular layers; G: granular layer; IG: infragranular layers.

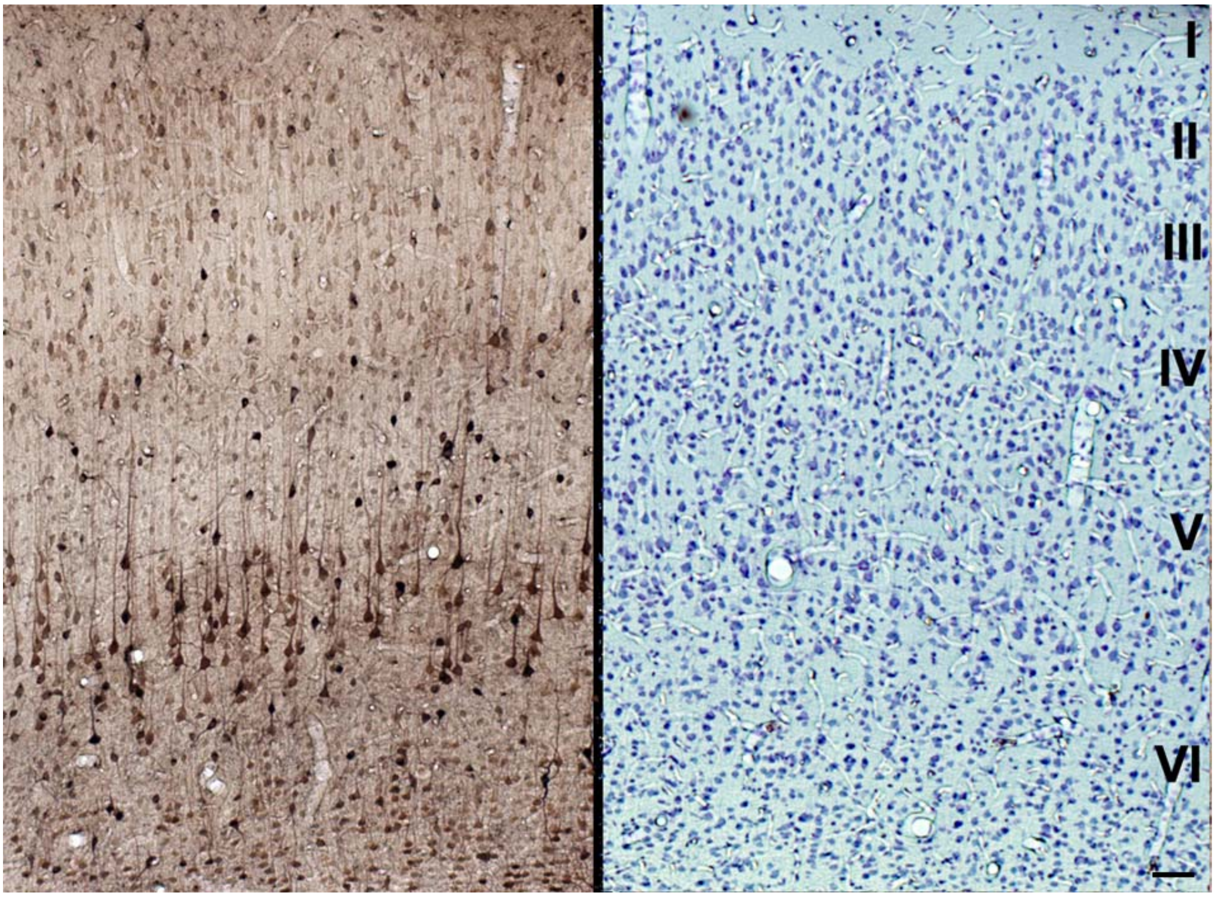


*Supplementary figure 3.* Photomicrographs of PtA stained for $Tl^+$ (left, WK) and Nissl (right). Note that layer IV is sparse and hard to delineate in this cortical field. Scale bar is 50 µm.

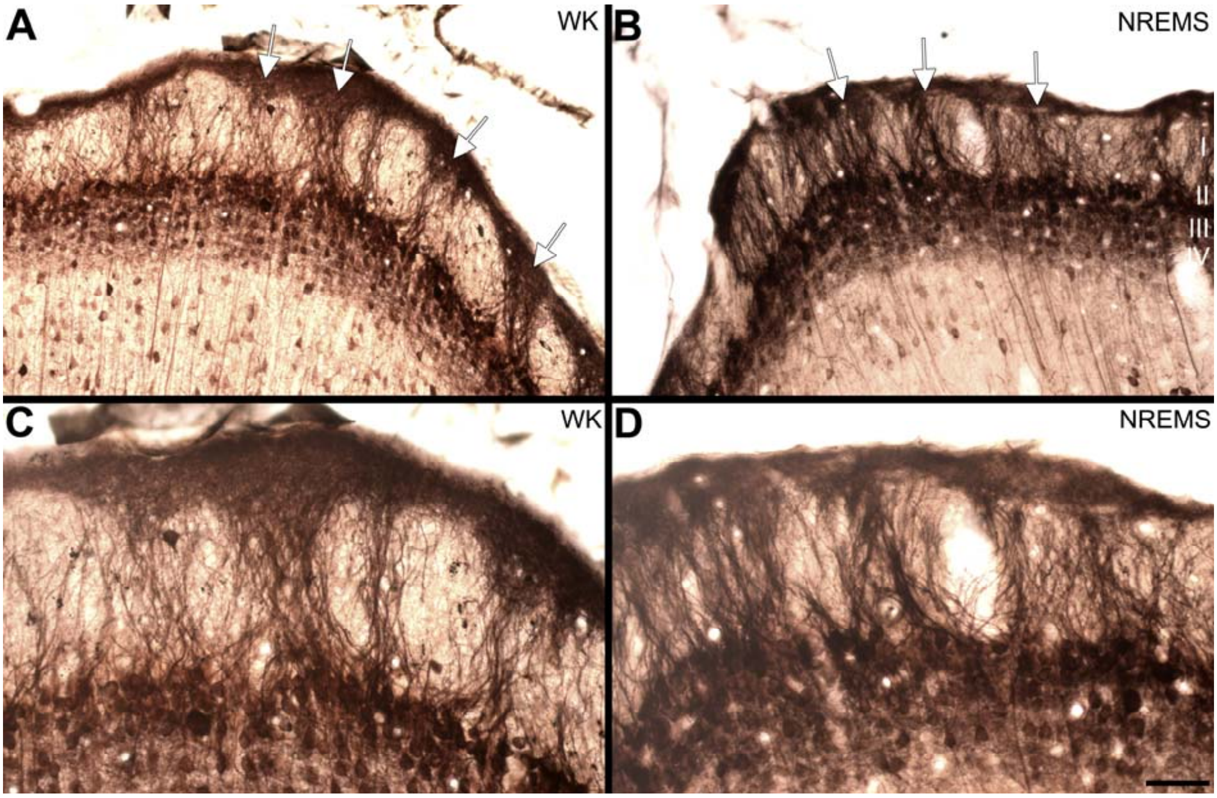


*Supplementary figure 4.* $Tl^{+}$-uptake in rat retrosplenial association cortex, granular part (RSG). Shown are photomicrographs from superficial layers of RSG (Bregma level. approx. -3.80) for a WK animal (A and C), and a NREMS animal (B and D). Panels C and D show details of panels A and B. Note intense staining of dendritic clusters in RSG layer I (arrows in A and B) in both animals. Shape and size of dendritic staining clusters bear resemblance to anatomically defined apical dendritic bundles of RSG layer II pyramidal cells (Wyss et al. J Comp Neurol. 1990 May 1;295(1):33-42). Note that the anteroventral thalamic nucleus has topographically selective projections onto these dendritic bundles (Wyss et al. 1990; Vogt & Vogt. Cingulate Cortex and Disease Models. In: The Rat Nervous System, Third Edition. 2004. Academic Press. p. 705 – 728). Scale bar is 100 µm in A and B, and 50 µm in C and D.

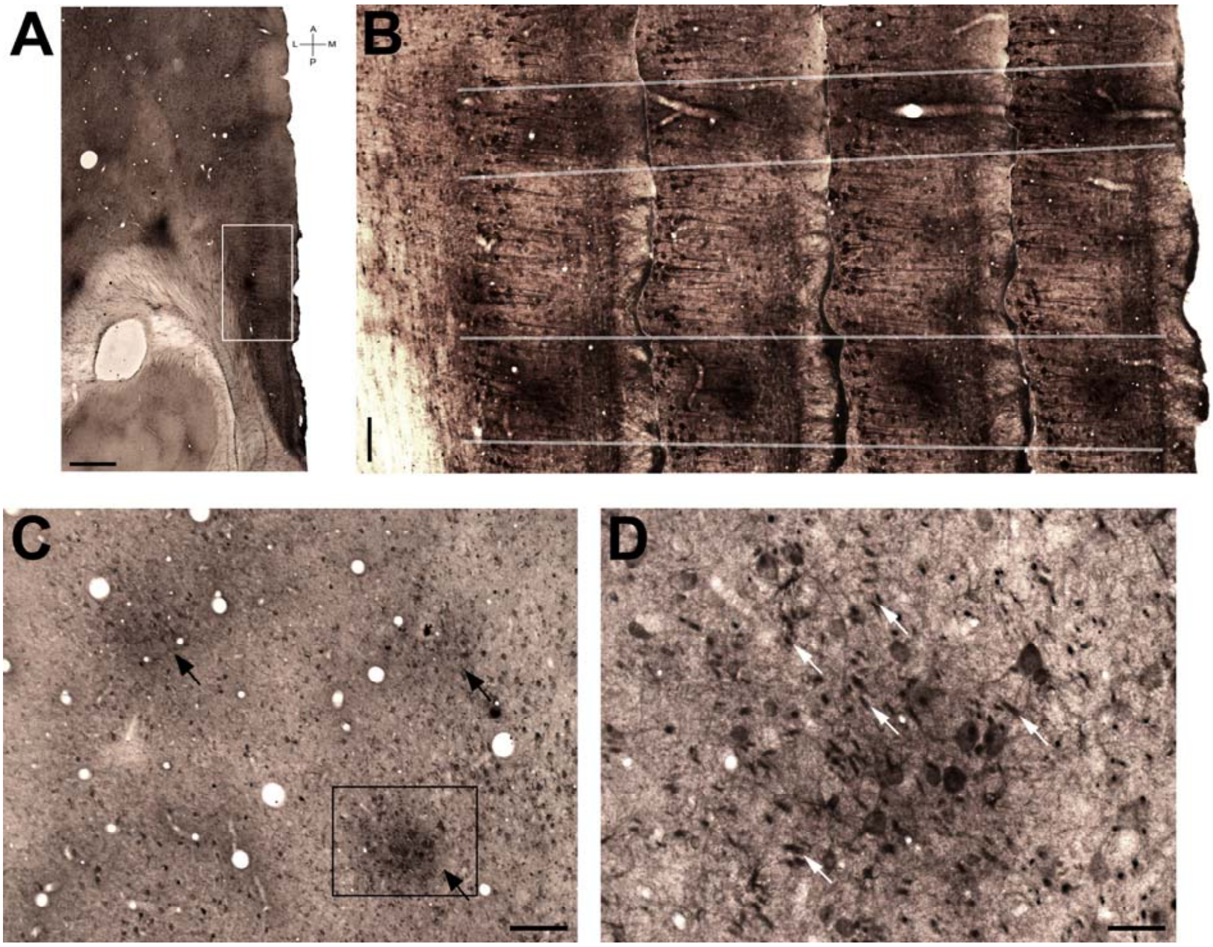


*Supplementary figure 5.* Columnar $Tl^+$-uptake patterns in NREMS as seen in horizontal sections. (A) Low magnification image of the medial part of a horizontal hemisection from a NREMS animal (approximately 3 mm below Bregma). The white rectangle indicates part of the ventral granular retrosplenial association cortex (RSG) as is shown in B. (B) Series of higher magnification images from consecutive horizontal hemisections as indicated in A from dorsal (left) to ventral (right). Semitransparent white lines outline columnar uptake patterns over consecutive sections. (C) High magnification image from the same animal as in A and B (approximately 1.5 mm below Bregma), supposedly showing the superficial layers of somatosensory or motor cortex. Note localized patches or clusters of comparatively high $Tl^+$-uptake (black arrows). The black rectangle indicates the position of D. (D) Detail as indicated in C. Note intensely stained neuronal cell bodies. Furthermore, note putative apical dendritic trunks from pyramidal cells located in deep layers which were cut diagonally (white arrows). Scale bar is 500 µm in A, 125 µm in B and C, and 31.25 µm in D.

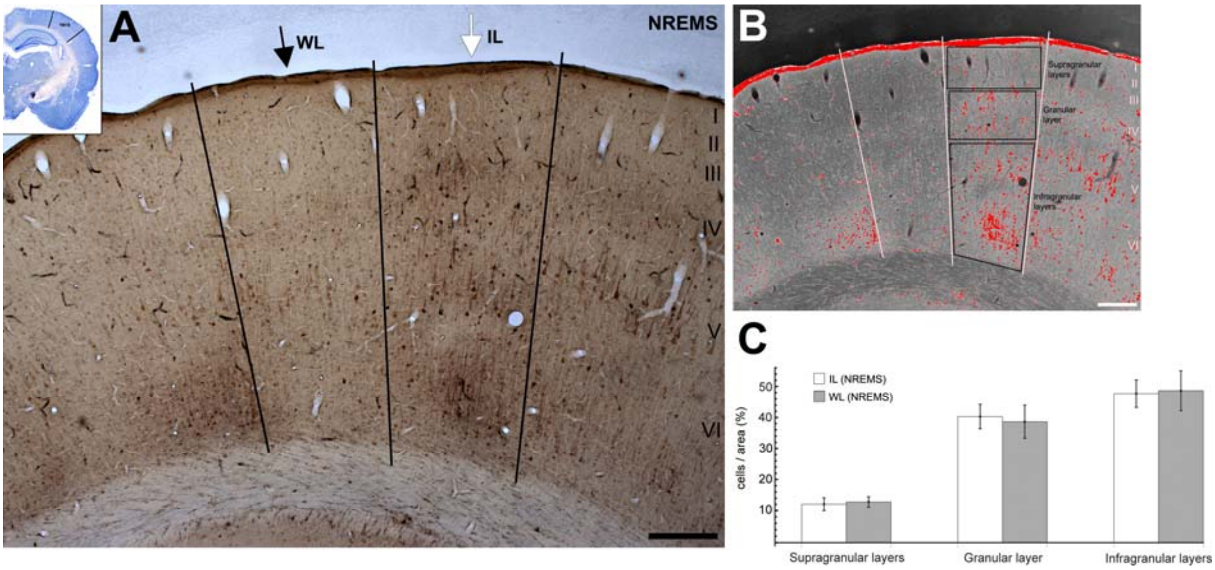


*Supplementary figure 6.* Comparison of laminar $Tl^+$-uptake for intensely labeled (IL) and weakly labeled (WL) columns in NREMS. (A) Photomicrograph of somatosensory cortex illustrating staining patterns in intensely (white arrow) and weakly (black arrow) labeled columns as indicated by black lines. The inset indicates the position of the exemplary photomicrograph in a neighboring Nissl section. Scale bar is 250 µm. (B) Inverted gray scale image of the photomicrograph shown in A. Pixels were thresholded via ImageJ (NIH) and cells with gray values above threshold were counted using the built-in function “Analyze Particles” (see Methods). Black polygons indicate ROIs for supragranular, granular and infragranular layers, respectively. (C) Statistical analysis of laminar differences in cellular $Tl^+$-uptake. Analyses were performed on 10 – 15 intensely and weakly labeled columns, respectively, from different cortical areas (between Bregma -2.30 and -5.80) for each NREMS-animal (n = 6). Percentage figures indicate the ratio of suprathreshold cells in each laminar ROI to the overall number of suprathreshold cells in the corresponding column. Data are means ± SEM. Statistical significance of group differences was evaluated with a signed-rank test ($p > 0.80$ for every ROI).

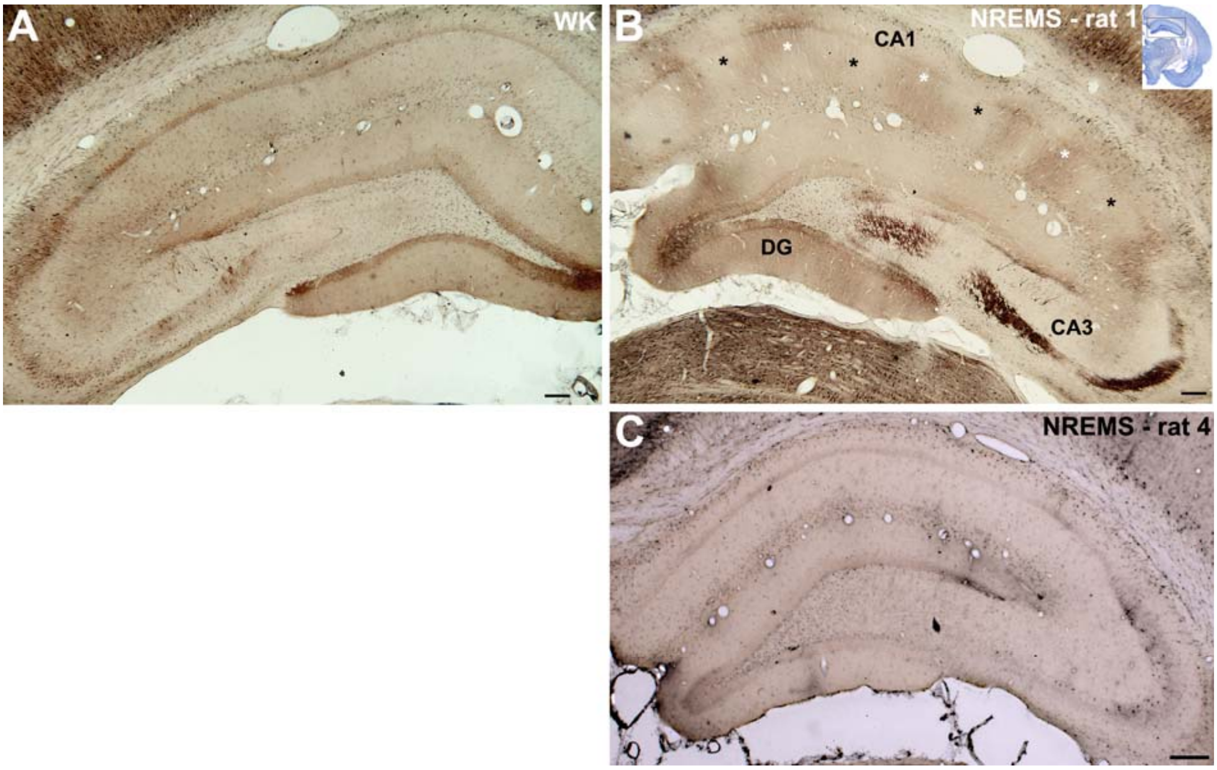


*Supplementary figure 7.* Hippocampal $Tl^+$-uptake patterns in WK and NREMS. Shown are photomicrographs from one WK animal (A) and two NREMS animals (B and C). The inset in B indicates the rostrocaudal positions of the photomicrographs in a frontal overview of a Nissl section. Note alternating clusters of high $Tl^+$-uptake (white asterisks) in CA1 stratum radiatum and stratum pyramidale, adjacent to regions where $Tl^+$-uptake is comparably low (black asterisks) in B, but not in A and C. Overall, columnar uptake patterns were salient in three of the six NREMS animals, but were not observed in any of the five WK animals. Scale bar is 200 µm in A and B, and 250 µm in C.

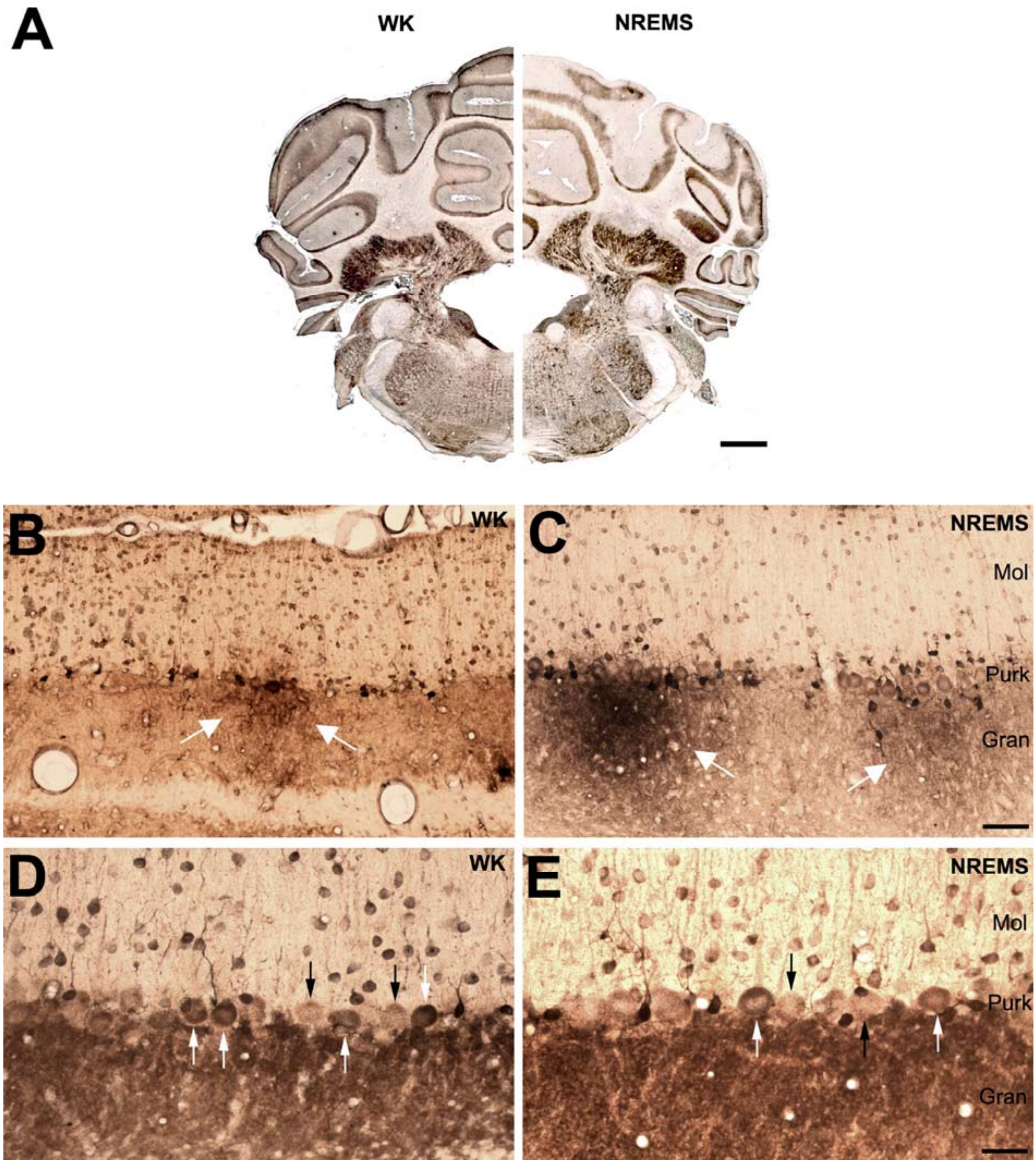


*Supplementary figure 8.* $Tl^+$-uptake in rat cerebellar cortex for WK and NREMS. (A) Frontal hemisections on the level of the rostral cerebellum arranged as mirror images for WK (left) and NREMS (right). (B) and (C) $Tl^+$-uptake clusters in cerebellar granular layer and Purkinje cell layer as indicated by arrows for both WK and NREMS. Note that, in general, $Tl^+$-uptake clusters appeared to be less abundant in cerebellar cortex as compared to the cerebral cortex. (D) and (E) Detailed photomicrographs of cerebellar Purkinje cell layer. Note intensely stained Purkinje cell bodies (white arrows) adjacent to comparatively weakly

stained Purkinje cell bodies (black arrows). Abbreviations: Gran: granular layer; Mol: molecular layer; Purk: Purkinje cell layer. Scale bar is 1 mm in A, 62.5 μm in B and C, and 31.25 μm in D and E.

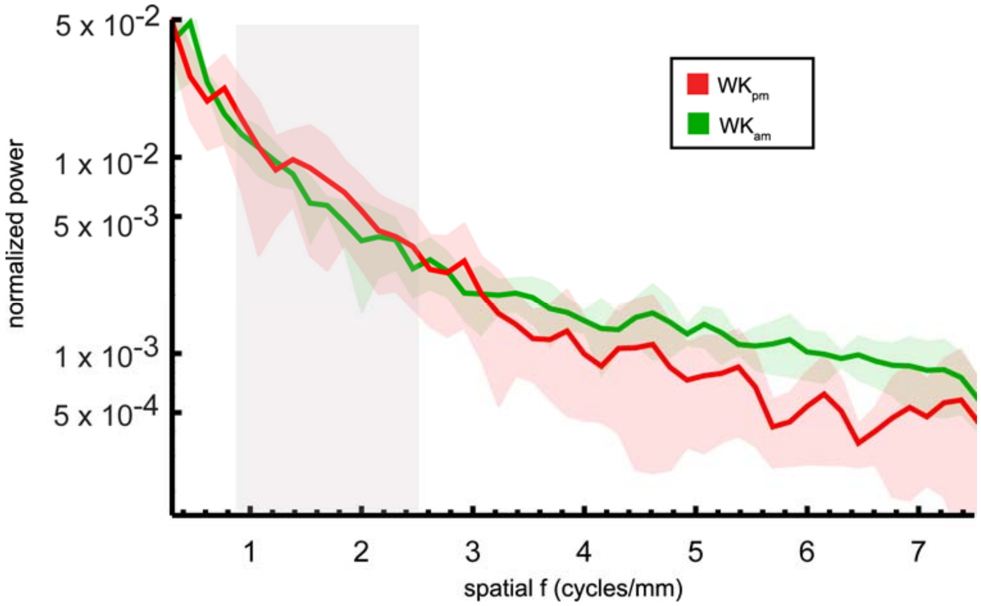


*Supplementary figure 9.* Quantification of spatial periodicities in cortical $Tl^+$-uptake. Analyses were performed on inverted gray scale images of scanned frontal sections (15 - 20 per animal) from caudal neocortex (Bregma -5.60 to -6.30) for wakefulness in the evening (between 6pm and 8pm, $WK_{pm}$) and in the morning (between 9am and 11am, $WK_{am}$). ROIs included the whole cortical mantle from the lateral edge of retrosplenial areas to the ventral edge of the perirhinal cortex. Shown are grand averages of normalized PSD plots (semi-logarithmic scale, n = 5 for $WK_{pm}$, n = 4 for $WK_{am}$). Data are means (thick lines) ± standard deviations of the mean. The gray shaded area (0.83 – 2.5 cycles/mm) indicates the spatial frequency bin that corresponds to reported diameters for cortical macrocolumns (200 – 600 µm). Analysis of individual PSD mean value pairs within the indicated spatial frequency bin revealed no statistically significant differences between $WK_{pm}$ and $WK_{am}$ (two-tailed Mann-Whitney U-test).
